# The Search for Biomarkers to Aid in Diagnosis, Differentiation, and Prognosis of Childhood Idiopathic Nephrotic Syndrome

**DOI:** 10.3389/fped.2019.00404

**Published:** 2019-10-16

**Authors:** Hillarey Stone, Bliss Magella, Michael R. Bennett

**Affiliations:** ^1^Division of Nephrology and Hypertension, Cincinnati Children's Hospital Medical Center, Cincinnati, OH, United States; ^2^Department of Pediatrics, University of Cincinnati College of Medicine, Cincinnati, OH, United States

**Keywords:** nephrotic syndrome, steroid resistance, biomarkers, focal segmental glomerulosclerosis, minimal change disease

## Abstract

Identification of genes associated with childhood-onset nephrotic syndrome has significantly advanced our understanding of the pathogenesis of this complex disease over the past two decades, however the precise etiology in many cases remains unclear. At this time, we still rely on invasive kidney biopsy to determine the underlying cause of nephrotic syndrome in adults. In children, response to steroid therapy has been shown to be the best indicator of prognosis, and therefore all children are treated initially with corticosteroids. Because this strategy exposes a large number of children to the toxicities of steroids without providing any benefit, many researchers have sought to find a marker that could predict a patient's response to steroids at the time of diagnosis. Additionally, the identification of such a marker could provide prognostic information about a patient's response to medications, progression to end stage renal disease, and risk of disease recurrence following transplantation. Major advances have been made in understanding how genetic biomarkers can be used to predict a patient's response to therapies and disease course, especially after transplantation. Research attempting to identify urine- and serum-based biomarkers which could be used for the diagnosis, differentiation, and prognosis of nephrotic syndrome has become an area of emphasis. In this review, we explore the most exciting biomarkers and their potential clinical applications.

## Introduction

Nephrotic syndrome (NS) is the most common glomerular disease of childhood, with an annual incidence of between 1 and 17 per 100,000 children, depending on the population (1, 2). Though morbidity and mortality has improved significantly with the use of corticosteroids, children with nephrotic syndrome remain at risk for life-threatening infections, venous thromboembolism, dyslipidemia, and chronic kidney disease (3, 4). Approximately 80–90% of children with nephrotic syndrome achieve remission with a 4 weeks course of corticosteroids and are therefore given the diagnosis of steroid sensitive nephrotic syndrome (SSNS). When biopsied, these patients are more likely to have minimal change disease (MCD). Patients with SSNS tend to have an excellent overall prognosis, with <5% progressing to chronic kidney disease (4). The remaining 10–20% of patients have primary steroid resistant nephrotic syndrome (SRNS), are more likely to have focal segmental glomerulosclerosis (FSGS) on biopsy, and have up to a 50% risk of developing end stage renal disease (ESRD) within 5 years of diagnosis. Approximately 17–25% of patients who initially respond to steroids will go on to develop steroid resistance; this is defined as secondary SRNS (5, 6).

Invasive kidney biopsy remains the gold standard for diagnosing nephrotic syndrome, however routine biopsies are no longer performed at diagnosis in children, due to their invasive nature. With our current technology, a child's response to steroids has been shown to be the best indicator of overall prognosis (3, 7). Therefore, all patients are initially treated with high dose corticosteroids. This may unnecessarily expose patients with SRNS to the adverse effects of steroids and could delay treatment with other therapies to which they may be more likely to respond.

We currently have a dearth of clinical or laboratory biomarkers that can predict whether a patient will respond to steroids or other immunosuppressive therapies. What is desperately needed are non-invasive tests which could allow us to predict which patients will respond to steroids or to steroid-sparing immunosuppressive agents, and which patients should be treated supportively with anti-proteinuric medications. Recent and ongoing studies are attempting to identify biomarkers and genetic panels that could help with the diagnosis, discrimination, and prognosis of nephrotic syndrome in children.

While nephrotic syndrome is a heterogeneous disease with multiple potential underlying etiologies, a hallmark of all forms of nephrotic syndrome is effacement of the podocyte foot processes, which causes disruption of the glomerular filtration barrier and leads to massive proteinuria. The precise etiology underlying this damage is still not completely understood, though a great deal of progress has been made in this area over the past two decades. For example, it is now well-known that certain genetic variants lead to dysfunctional proteins which cause underlying structural abnormalities in the glomerular filtration barrier. Furthermore, there is good evidence to suggest that the immune system, and specifically T lymphocyte dysfunction, may be involved in other non-genetic forms of nephrotic syndrome. This is supported by the knowledge that nephrotic syndrome responds to corticosteroids and other immunosuppressive medications [reviewed by (8)], by cases of nephrotic syndrome resolving following measles infection (9), and by the association between T cell lymphoma and the diagnosis of nephrotic syndrome (10). Finally, it is theorized that a circulating glomerular permeability factor is responsible for some forms of nephrotic syndrome, though a specific putative factor has yet to be identified. This theory is supported by the recurrence of FSGS following kidney transplantation and the successful treatment of FSGS recurrence with immunoadsorption and therapeutic plasma exchange (11).

At this time, no validated biomarkers exist for the diagnosis, differentiation, or prognostication of idiopathic nephrotic syndrome. Here we review candidate urinary, serum, and genetic biomarkers which have been extensively researched and have potential for clinical application in the near future. While this is not an exhaustive list of biomarkers that have been studied in this disorder, it highlights those that are most promising for future clinical use.

## Urine and Serum Biomarkers

### Urinary Vitamin D Binding Protein

It is well-established that patients with nephrotic syndrome have low serum levels of vitamin D. This is primarily a result of loss of vitamin D binding protein which is seen in high levels in the urine during active nephrotic syndrome (12–14). Bennett et al. studied urinary vitamin D binding protein (uVDBP) levels in a cohort of children with nephrotic syndrome and found that uVDBP levels were significantly elevated in patients with SRNS when compared to patients with SSNS, both during remission and relapse. This finding remained significant even when controlled for proteinuria and estimated glomerular filtration rate (eGFR). Receiver operating characteristic (ROC) curve analysis resulted in an area under the curve (AUC) of 0.87 for the discrimination of SSNS vs. SRNS. They concluded that uVDBP could therefore be used as a non-invasive biomarker to predict steroid sensitivity in children diagnosed with idiopathic nephrotic syndrome (13). While the results were promising, this work was done in a small population at a single center, and to date no multicenter, prospective study has been attempted.

### Urine NGAL

Neutrophil gelatinase-associated lipocalin (NGAL) is constitutively expressed at low levels in a variety of human tissues including bone marrow, stomach, colon, lung, liver, and the kidney (15). Initial studies using animal models identified NGAL as an early marker of renal injury (16). During times of kidney injury, NGAL expression is upregulated in the renal tubules and therefore is a well-studied marker of acute and chronic kidney injury (17). NGAL has been shown to be a strong predictor of disease progression in patients with chronic kidney disease (18, 19). Nickolas et al. showed that participants had significantly elevated urine NGAL levels associated with biopsy confirmed interstitial fibrosis and tubular atrophy (19). In a recent pilot study, Bennett et al. measured urine NGAL levels in patients with SSNS and SRNS and in healthy controls with the goal to determine whether NGAL could predict steroid sensitivity. They found that urine NGAL levels were significantly higher in patients with SRNS compared to patients with SSNS and healthy controls. These findings remained significant when normalized for urine creatinine. ROC curve analysis was performed, and AUC to distinguish SSNS from SRNS was 0.91 (*p* < 0.0001). They found that NGAL did not correlate with proteinuria, though it was negatively correlated with eGFR (20).

NGAL is a well-studied and validated marker which already has a number of clinical applications in other kidney diseases. Initial studies suggest that it may also be useful for the prediction of steroid sensitivity in patients with nephrotic syndrome, however it has yet to be validated in a large cohort of patients with this disease. Additional work will also be needed to understand the effects of abnormal glomerular filtration rate (GFR) and degree of proteinuria on the utility of NGAL in this context.

### α 1-B Glycoprotein

α 1-B glycoprotein (A1BG) is a member of the immunoglobulin superfamily, although its function is currently unknown. Piyaphanee et al. used surface-enhanced laser disruption/ionization time of flight mass spectrometry (SELDI-TOF-MS) to examine the urine of patients with idiopathic nephrotic syndrome and healthy controls and found that a fragment of A1BG was only present in patients with nephrotic syndrome. Furthermore, a 13.8 kilodaltons (KDa) A1BG fragment was detected in patients with SRNS but not in patients with SSNS (21). Though these results were significant, no further studies have attempted to validate this as a biomarker to discriminate SRNS from SSNS. However, one can imagine the possible utility of this biomarker in the clinical setting. Utilizing western blot technology, clinical labs could determine the presence and fragment size of A1BG in urine samples. This technique would allow for the incorporation of A1BG into future biomarker panels that will be used to identify a patient's likely diagnosis and treatment plan. Future studies are needed to validate this biomarker in a larger patient cohort, either alone or as part of a panel of biomarkers.

### CD80 (B7-1)

CD80, also known as B7-1, is a transmembrane protein which is expressed on the surface B cells and other antigen presenting cells. Once stimulated, it leads to activation of T cells via CD28 or inactivation of T cells via cytotoxic T-lymphocyte-associated protein 4 (CTLA4). In podocytes, its activation causes reorganization of the actin cytoskeleton, effacement of foot processes, and ultimately proteinuria (22, 23). Garin et al. showed that urinary CD80 levels were significantly elevated in patients with MCD (*n* = 19) during relapse compared with those in remission (24). The same group went on to show that urinary CD80 was elevated in the urine of patients with relapsed MCD (*n* = 17) compared to patients with MCD in remission or patients with FSGS (*n* = 22). ROC curves were used to compare CD80 levels in relapsed MCD vs. FSGS and for MCD in relapse vs. MCD in remission, and the AUCs were 0.99 and 1.00, respectively. The authors concluded that CD80 could be a valuable marker for distinguishing MCD from FSGS (25). Ling et al. confirmed these conclusions in 2015, when they also showed that urine CD80 levels were higher in patients with active MCD compared to patients with MCD in remission, patients with FSGS or other glomerulopathies, and healthy controls. They went on to use ROC curves to assess the ability of CD80 to discriminate MCD (*n* = 37) from FSGS (*n* = 27), and found an AUC of 0.925, sensitivity 81.1%, and specificity 94.4% (22). Several other research teams have found similar differences in urine CD80 when comparing relapsed MCD, remission MCD, and FSGS (26–29).

CD80 is of particular interest, as a therapeutic agent exists which is targeted against this protein. Abatacept (CTLA-4-Ig) is a CD80 inhibitor which is FDA approved for the treatment of rheumatoid arthritis. It has been used off-label for other autoimmune diseases as well as in some cases of nephrotic syndrome. Yu et al., described a series of five patients with FSGS whose podocytes stained positive for CD80 and who had complete or partial remission of proteinuria following treatment with abatacept (30). Unfortunately, since these initial findings were published, many groups have expressed concern about the reliability of CD80 immunohistochemical assays and have been unable to replicate the original findings by Yu and colleagues. Novelli et al., studied CD80 expression in both patients with MCD (*n* = 15) and FSGS (*n* = 16) compared with healthy controls and in mice with adriamycin-induced nephropathy, and found no upregulation of B7-1 expression in podocytes in humans or in their animal models (31). Garin et al., described five patients with MCD or primary FSGS who were treated with abatacept. They reported a dramatic decrease in urinary CD80 levels following administration of abatacept in a patient with MCD, as well as transient improvement of proteinuria. They did not see similar improvement in proteinuria in the patients with FSGS (32). Several other groups attempted to replicate the work by Yu et al., but were unsuccessful. Some propose that because Yu et al. did not use a negative control during their staining procedure, their findings may have simply represented artifact (33–35).

CD80 has been extensively studied, both *in vitro* and *in vivo*, and is one of the most exciting candidate biomarkers for nephrotic syndrome to date. While validation in large patient cohorts is still needed, this could be a useful marker for both prognostication and to guide personalized treatment approaches.

### A Promising Urine Panel for the Discrimination of SSNS and SRNS

Bennett et al. studied the urine proteome in patients with steroid sensitive (*n* = 25) and SRNS (*N* = 25). Using isobaric tags for relative and absolute quantitation (iTRAQ), they found 13 proteins that were significantly different between the two groups (SSNS vs. SRNS). They went on to use a panel of 10 biomarkers [alpha-1 acid glycoprotein, alpha-1 acid glycoprotein 2, alpha-1 microglobulin, alpha-1-B glycoprotein, fetuin-A, hemopexin, NGAL, prealbumin (transthyretin), thyroxine-binding globulin, and VDBP] and found that the panel was able to predict SSNS vs. SRNS better than any one biomarker alone, with an AUC of 0.92 (36). Future studies are needed to validate this panel in a larger, multi-center cohort.

## Serum based Biomarkers

### Circulating Permeability Factors

In the 1950s, Gentili et al., set out to test the theory that idiopathic nephrotic syndrome was caused by a circulating glomerular permeability factor by transfusing blood from patients with nephrotic syndrome to healthy, non-nephrotic patients (37). Since that time, an explosion of research has ensued in attempt to identify a specific circulating factor that could be responsible for most or all cases of non-genetic nephrotic syndrome. Identifying a culpable circulating factor may be the Holy Grail in the world of NS biomarker research, as this could be used as a prognostic marker as well as a therapeutic target.

Many observations support the theory that a circulating factor may be responsible for at least some forms of SRNS. First, it is well-known that proteinuria often recurs quickly following transplant in a subset of patients, and many of these patients respond fully or partially to plasmapheresis (11). Second, experiments in animals have shown that when rats are exposed to plasma from patients with FSGS they develop proteinuria (38). Finally, case reports have described transmission of FSGS from pregnant women to their newborn infants (39, 40) and FSGS recurrence developing (both clinically and histologically) in a transplanted kidney and then resolving when the graft was removed and transplanted into a new recipient without history of FSGS (41).

Those with presumed circulating factor disease are a particularly challenging subset of patients, who often develop very early recurrence of nephrotic syndrome following transplant. The identification of a measurable factor could guide initial management, allow for improved prediction of post-transplant recurrence, and potentially allow clinicians to develop more tailored peri-transplant immunosuppressive protocols for patients with circulating factor disease. The hunt for a specific circulating factor has been the subject of a great deal of research in the past 50 years, and the most exciting progress will be reviewed here.

### Hemopexin

Hemopexin is a heme scavenger which is produced by the liver and acts as an acute phase reactant in response to infection or inflammation. Hemopexin was first identified as a potential circulating permeability factor in the late 1990s and received a great deal of attention early on in the hunt for a putative permeability factor (42). *In vitro* incubation of kidney tissue with hemopexin led to loss of glomerular sialoglycoproteins and ectoapyrase, and this effect was inhibited by the addition of a serine protease inhibitor (phenylmethanesulfonyl fluoride) (43). Hemopexin infused into rats led to increased proteinuria and foot process effacement (43, 44). *In vitro* treatment of human kidney tissue with hemopexin caused alterations in the components of the glomerular filtration barrier, including reorganization of the actin cytoskeleton (45). In a small study evaluating 41 children with MCD, lower levels of plasma hemopexin were observed in patients in relapse compared to those in remission and in controls. In this same group, an increase in hemopexin activity was observed in patients during relapse (46). Despite these exciting early findings, hemopexin has lost steam as a potential circulating factor and biomarker. It has yet to be studied in a large cohort of patients with nephrotic syndrome. Furthermore, it is unclear as to what factors stimulate the production of hemopexin, and whether its activation is caused by or a result of the immunologic derangements underlying the development of nephrotic syndrome.

### Soluble Urokinase Plasminogen Activator Receptor (suPAR)

Urokinase plasminogen activator receptor (uPAR) and its soluble form suPAR are perhaps the most widely studied of the potential circulating factor candidates. uPAR is a glycosylphosphatidylinositol (GPI)-anchored cell membrane receptor that binds urokinase plasminogen activator (uPA). This receptor is expressed on a variety of cells, including T cells, neutrophils, macrophages, smooth muscle cells, and endothelial cells, and it is involved in a number of cell functions including adhesion, proliferation, cell survival, and inflammation (47). When its GPI anchor is cleaved, this protein can be released from the plasma membrane as soluble urokinase plasminogen activator (suPAR) which then acts on receptors including β3 integrin on podocytes. suPAR has been extensively studied as a biomarker in many inflammatory diseases (sepsis, inflammatory bowel disease, systemic lupus erythematosus, heart disease, etc.) and cancers (47).

suPAR first gained attention for its potential role in nephrotic syndrome in 2001, when Xu et al. showed that uPAR was expressed in glomerular cells during nephritis and proposed that this could lead to development of glomerulosclerosis (48). In 2008, Wei et al. showed that overexpression of uPAR led to effacement of podocyte foot processes and proteinuria in mice and therefore proposed that suPAR could be a potential circulating factor in nephrotic syndrome (49). In 2011, the same group measured serum suPAR levels in patients with FSGS (*n* = 78), other glomerular diseases (*n* = 48), and healthy controls (*n* = 22) and found elevated levels in patients with FSGS but not in those with MCD, membranous nephropathy, or preeclampsia. Furthermore, they showed that patients had decreased suPAR concentrations following plasmapheresis, suggesting that plasmapheresis could be effective in clearing this molecule and further supporting their hypothesis that this could be the circulating factor responsible for FSGS (50). The same group measured suPAR levels in two large FSGS cohorts (*n* = 165)—the FSGS clinical trial (FSGS-CT) group, which consisted of children and adults with primary FSGS, and the consortium for study of steroid-resistant nephrotic syndrome (PodoNet) cohort, which was composed of patients with primary, childhood-onset SRNS, congenital nephrotic syndrome, or presumed genetic proteinuria without clinical nephrotic syndrome. They again showed that patients with primary FSGS had elevated serum suPAR concentrations. Moreover, they showed that suPAR levels in these patients could not be explained by inflammation, as c reactive protein (CRP) levels were not elevated (51). These exciting findings led to an explosion of work in this area, and many groups attempted to replicate these results in human and animal models (52–55).

Cathelin et al., used a mouse model to study the effect of suPAR on podocytes and found that though suPAR was deposited in the glomeruli, this did not lead to structural changes in podocytes or proteinuria (54). Spinale et al. attempted to replicate Wei's findings by injecting wild type and transgenic mice with suPAR, however they were unable to induce proteinuria. The same group also measured suPAR levels in 241 patients enrolled in the Nephrotic Syndrome Study Network (NEPTUNE) and found that suPAR concentrations correlated with degree of proteinuria and estimated GFR but not with histologic diagnosis. Specifically, patients with FSGS were not found to have higher suPAR levels than those with MCD or IgA nephropathy (55).

Several other studies have failed to replicate the initial results of Wei et al. and suggest that suPAR is not by itself responsible for FSGS, nor can it reliably be used to discriminate steroid sensitive from SRNS. Many studies have, however, demonstrated that suPAR is inversely correlated with estimated GFR, both in adults (52) and children (53, 56, 57). This could suggest that suPAR is filtered by healthy glomeruli, and therefore decreased GFR leads to elevated serum levels. Other studies suggest that suPAR may simply be a marker of inflammation. Clearly, further investigations are needed to better define suPAR's role as a marker of chronic kidney disease, its specific role in inflammation, and whether it could be involved in a “two-hit” model of FSGS.

### Cardiotrophin-Like Cytokine Factor 1 (CLCF-1)

CLCF-1 is a cytokine in the interleukin 6 (IL-6) family. It is expressed in a number of tissues, including bone marrow, lymphocytes, lymph nodes, spleen, and kidney, and is known to activate B cells (58, 59). CLCF-1 has been shown to activate the JAK/STAT pathway in a number of cell types. In an *in vitro* study, CLCF-1 led to increased glomerular albumin permeability, and this increase was inhibited by anti-CLCF-1 monoclonal antibody. Chronic administration of CLCF-1 in mice led to focal glomerular scarring, and therefore it was proposed that it may play a role in the development of FSGS in humans (58). CLCF-1 has also been shown to bind to ApoE, a molecule involved in lipid metabolism, as well as with lipoproteins low density lipoprotein (LDL) and very low density lipoprotein (VLDL). Based on these findings and the fact that LDL pheresis can be used in some cases of FSGS, CLCF-1 has been proposed as a potential circulating factor in the pathogenesis of FSGS (60). CLCF-1 is present in the plasma of patients with FSGS and is elevated in patients with recurrent FSGS when compared to healthy controls (61). CLCF-1 is promising in the hunt for a circulating permeability factor, and therefore could potentially be used as a serum based biomarker. However, to date it has not been studied in large patient cohorts.

### Angiopoietin-Like Factor 4 (Angptl4)

Angiopoietin-like-4 (Angptl4) is a protein involved in triglyceride clearance and lipid metabolism. It is highly expressed in liver and adipose tissue and is upregulated in podocytes in experimental models of glomerular injury (62). In a puromycin aminonucleoside nephrosis (PAN) model, *Angptl4* expression was upregulated in rat glomeruli, an effect that was observed even prior to the development of proteinuria (62). *Angptl4* transgenic rats showed increased glomerular expression of Angptl4, foot process effacement, and selective proteinuria when compared to wild-type littermates. Subsequent treatment of these rats with corticosteroids led to improvement in proteinuria and a significant decrease in *Angptl4* expression (62). Similar results were found in an adriamycin-induced rat model of MCD. Upregulation of glomerular Angptl4 was observed in the adriamycin treated rats when compared to normal rats. Again, this effect was apparent before the development of proteinuria, suggesting that Angptl4 could be an early marker of podocyte injury. Treatment with tacrolimus led to decreased glomerular Angptl4 expression and improvement in urinary Angptl4 excretion (63).

Though initial observational and animal model studies supported Angptl4 as a potential key player in the development of nephrotic syndrome and therefore a reasonable candidate biomarker, evaluation in patients with nephrotic syndrome has yielded conflicting results. In a small study including five patients with MCD and an unspecified number of controls, Angptl4 expression was increased in patient kidney biopsy tissue. This increased expression was noted to be in a podocyte-specific distribution (62). Another study compared Angptl4 expression in kidney tissue of 30 adults with MCD, FSGS, membranous nephropathy (MN), and mesangial proliferative glomerulonephritis (MsPGN). Angptl4 expression was upregulated in patients with MCD compared to those with mesangial proliferative glomerulonephritis, and urinary Angptl4 excretion was higher in MCD, MN, and FSGS patients compared to those with MsPGN, however no differences were observed in patients with MCD vs. FSGS or MN (63). Another study which included 60 children and adults with MCD, 52 adults with FSGS, and 18 controls examined Angptl4 in urine, serum, and kidney tissue. Urinary Angptl4 was elevated in all patients with significant proteinuria, regardless of the underlying cause. Serum levels were not significantly different in patients during relapse vs. remission or in relapsed disease compared to controls. Glomerular Angptl4 was not expressed in glomeruli of patients with MCD in relapse, while it was variably expressed in other disease states (64). Overall, Angptl4 seems to be an early marker of podocyte injury, however its role in distinguishing MCD from other nephropathies has yet to be defined. Further studies evaluating Angptl4 levels in serum and urine in a larger, more homogenous group of patients is needed before this could be recommended as a clinical biomarker for nephrotic syndrome.

### CD40 and Anti-CD40 Antibodies

CD40 is a member of the tumor necrosis factor (TNF) superfamily and is involved in the adaptive immune response (65). It is expressed on B lymphocytes, macrophages, dendritic cells, and other antigen presenting cells. In the kidney, CD40 is expressed in mesangial, tubular, and glomerular epithelial cells. When stimulated, it leads to a pro-inflammatory response in mesangial and tubular cells, while its specific role in podocytes is less clear. Interestingly, its activation leads to increased synthesis of suPAR by endothelial cells. Delville et al., examined the serum of 33 patients with recurrent FSGS following renal transplantation and found that pre-transplant elevation of anti-CD40 could predict post-transplant recurrence with 78% accuracy (66). This exciting finding led to several *in vivo* and *in vitro* studies examining CD40's structural effects on podocytes and its potential role in the development of proteinuria. Doublier et al., showed that soluble CD40 ligand (sCD40L) caused increased glomerular permselectivity in rat glomeruli, though they did not see significant increases in proteinuria after *in vivo* injection of sCD40L in mouse models. The same group went on to show that serum sCD40L levels are elevated in a cohort of patients with steroid-resistant or steroid-dependent NS compared with healthy controls, as well as in patients with FSGS compared to healthy subjects (65). While these results were exciting, this was a fairly small sample of patients (96 total), and the group was quite heterogeneous, including patients with steroid dependent NS, steroid resistant NS, congenital NS, and idiopathic membranous nephropathy. Further studies are needed to evaluate sCD40L levels in a larger cohort of patients with SRNS.

CD40 is a promising marker, as several potential therapeutic agents are currently under investigation. ASKP1240, or bleselumab, is a fully human anti-CD40 monoclonal antibody which is being examined in the setting of a number of different diseases. ASKP1240 has been studied for the treatment of psoriasis and other autoimmune diseases and for immunosuppression following kidney transplant (67, 68). Additionally, an ongoing clinical trial is looking at bleselumab for the prevention of FSGS recurrence after transplant (69). A number of other CD40 and CD40L targets are currently being evaluated in clinical trials [reviewed by (70)]. It seems that CD40 is another candidate marker which could be useful for both prognostication and as a therapeutic target. Further studies are needed to examine its usefulness as a clinical biomarker in large patient cohorts. A summary of urine and serum biomarkers and their relationship to nephrotic syndrome can be found in Table 1.

**Table 1:** Urine and serum based biomarkers and their correlation to nephrotic syndrome subtypes.

**Biomarker**	**Disease state with high levels**	**Disease state with low levels**	**Healthy controls**	**ROC AUC**
CD80 (22)	MCD- active	FSGS- remission MCD	Present at low levels	0.925
NGAL (20)	SRNS	SSNS	Present at low levels	0.91
uVDBP	SRNS	SSNS	Present at very low levels	0.87
A1BG (21)	Full size and truncated protein—SRNS	Full size protein—SSNS	Absent	
suPAR (51)	FSGS	MCD	Present at low levels	
Hemopexin (46)	MCD- remission	MCD- relapse	Present at high levels	
CLCF-1 (61)	FSGS- recurrence	FSGS	Present at low levels	
CD40 (65)	FSGS- recurrence	FSGS	Present at low levels	
Angptl4 (64)	Heavy proteinuria, regardless of underlying cause	Remission states with low to no proteinuria	Present at low levels	

### Genetic Testing as a Biomarker for Nephrotic Syndrome

The discovery of monogenic causes of nephrotic syndrome has advanced our understanding of the pathogenesis of NS and the role of the podocyte in this complex disease [reviewed by (71)]. The glomerular filtration barrier is composed of the fenestrated endothelial cells, the glomerular basement membrane (GBM), and the epithelial podocytes. Together, these structures form a charge- and size-selective barrier, which when defective can lead to massive proteinuria [(71), comments by (4), reviewed by (72)].

More than 50 monogenic causes of SRNS have been identified to date. Most of these genes encode proteins involved in the structure of the glomerular filtration barrier, specifically in the slit diaphragm and the podocyte actin cytoskeleton. Other genes identified in SRNS cases encode for mitochondrial proteins, nuclear transcription factors, and proteins involved in adhesion of the GBM to the podocyte (71, 73, 74). Identification of an underlying genetic cause of SRNS often has significant clinical implications, as it can help predict the response to corticosteroids and other immunosuppressive medications, determine the need to screen for extra-renal manifestations, provide information used to counsel family members about their risk, and predict recurrence of disease following kidney transplantation (4, 72, 73).

Currently no guidelines exist for the use of genetic testing in SRNS, though most experts agree that genetic testing should be performed in patients presenting with NS before age 1, in patients with family history of SRNS, and in patients with syndromic features [reviewed by (72)].

With the development of improved technology and decrease in cost of next generation testing, genetic testing has become more widely used in clinical practice. Currently the two most commonly used methods for genetic testing in nephrotic syndrome are targeted sequencing of candidate genes using a symptom-driven gene panel and whole exome sequencing (WES). Gene panels employ high-throughput polymerase chain reaction (PCR) amplification and sequencing to analyze several genes at once. At this time, this method is typically less labor-intensive and more cost-effective than WES. Targeted gene sequencing has relatively high yield, and its results are easy to understand. Targeted gene sequencing will not, however, identify novel mutations in preselected genes or within unpredicted genes. Therefore, this test has lower sensitivity in disorders with yet undiscovered genetic causes. The major benefit of WES is that it can identify known genetic causes as well as detect novel genetic associations. At this time, WES is more expensive and more labor-intensive. Additionally, WES often produces variants of uncertain clinical significance which require interpretation by a highly skilled individual and can detect pathogenic variants which are unrelated to the disorder for which the test was ordered, thus leading to ethical conundrums (71, 72). As more and more genes associated with SRNS are identified, and as next generation sequencing becomes more widely available and cost-effective, WES may take over as the go-to test for identifying a Mendelian cause in patients with SRNS.

### Understanding the Prevalence of Monogenic Nephrotic Syndrome

At this time, few large, multi-ethnic studies exist to examine the prevalence of genetic SRNS. Several small studies have resulted in a broad range of prevalence rates, likely due to the effects of race, rates of consanguinity, number of familial cases included, number of SRNS-associated genes known at the time the study was performed, and method of testing. Current reports estimate that 2.9–30% of cases of SRNS have an underlying monogenic cause (6, 74–77).

### Genes and Their Role in the Glomerular Filtration Barrier

To date, over 50 genes have been identified which, when mutated, cause autosomal dominant or autosomal recessive nephrotic syndrome (76–132). Identification of these genes has improved our understanding of the role of the podocyte and slit diaphragm in the pathogenesis of nephrotic syndrome. The majority of the genes associated with nephrotic syndrome encode proteins that are essential in the structure of the slit diaphragm, the podocyte actin cytoskeleton, or the GBM. Other genes encode proteins involved in the co-enzyme Q biosynthesis pathway, nuclear proteins, or transcription factors. The genes currently known to be associated with nephrotic syndrome are listed in Table 2.

**Table 2:** Monogenic causes of steroid resistant nephrotic syndrome.

**Gene**	**Protein**	**Inheritance**	**References**
**SLIT DIAPHRAGM-ASSOCIATED PROTEINS**			
CD2AP	CD2-associated protein	AR	(78)
CRB2	Crumbs homolog 2	AR	(79)
FAT1	FAT tumor suppressor homolog 1	AR	(80)
KIRREL2	Neph3/Filtrin	AR	(81)
NPHS1	Nephrin	AR	(82)
NPHS2	Podocin	AR	(83)
PLCE1	Phospholipase C, epsilon 1	AR	(84)
TRPC6	Transient receptor potential cation channel, subfamily C, member 6	AD	(85)
**ACTIN CYTOSKELETON PROTEINS**			
ACTN4	Actinin, alpha 4	AD	(86)
ANLN	Anillin, actin binding protein	AD	(87)
ARHGAP24	Rho GTPase-activating protein 24	AD	(88)
ARHGDIA	Rho GDP dissociation inhibitor (GDI) alpha	AR	(89)
CUBN	Cubulin (intrinsic factor-cobalamin receptor)	AR	(90)
EMP2	Epithelial membrane protein 2	AD	(91)
INF2	Inverted formin, FH2, and WH2 domain containing	AD	(92)
KANK1	KN motif and ankyrin repeat domain containing protein 1	AR	(93)
KANK2	KN motif and ankyrin repeat domain containing protein 2	AR	(93)
KANK4	KN motif and ankyrin repeat domain containing protein 4	AR	(93)
MAGI2	Membrane associated guanylate kinase, inverted 2	AR	(94)
MYH9	Myosin heavy chain 9	AD	(95)
MYO1E	Homo sapiens myosin IE	AR	(96)
PODXL	Podocalyxin	AD	(97)
PTPRO	Protein tyrosine phosphatase receptor type O	AR	(98)
SYNPO	Synaptopodin	AD	(99)
**MITOCHONDRIAL PROTEINS**			
ADCK4	AarF domain containing kinase 4	AR	(100)
COQ2	Coenzyme Q2 4-hydroxybenzoate polyprenyltransferase	AR	(101)
COQ6	Coenzyme Q6 mono-oxygenase	AR	(102)
MTTL1	Mitochondrially encoded tRNA leucine 1	Mitochondrial	(103)
PDSS2	Prenyl diphosphate synthase subunit 2	AR	(104)
**GBM AND ADHESION PROTEINS**			
CD151	CD151 antigen	AR	(105)
COL4A3	α3 type IV collagen	AR	(106)
COL4A4	α4 type IV collagen	AR	(106)
COL4A5	α5 type IV collagen	XL	(107)
EXT1	Glycosyltransferase	AR	(108)
ITGA3	Integrin alpha 3 (antigen CD49C, alpha 3 subunit of VLA-3 receptor)	AR	(109)
ITGB4	Integrin beta 4	AR	(6, 110)
LAMA5	Laminin alpha5	AD/AR	(111)
LAMB2	Laminin β2	AR	(112)
**NUCLEAR PROTEINS AND TRANSCRIPTION FACTORS**			
E2F3	E2F transcription factor	AD	(113)
LMNA	Lamin A and C	AD	(114)
LMX1B	LIM homeobox transcription factor 1, beta	AD	(115, 116)
MAFB	Leucine zipper transcription factor	AD	(117)
NUP93	Nucleoporin 93 kDa	AR	(118)
NUP107	Nucleoporin 107 kDa	AR	(119)
NUP205	Nucleoporin 205 kDa	AR	(118)
NXF5	Nuclear RNA export factor 5	XL	(120)
PAX2	Paired box protein 2	AD	(121)
SMARCL1	SWI/SNF-related, matrix-associated, actin-dependent regulator of chromatin, subfamily a-like 1	AR	(122)
WDR73	WD repeat domain 73	AR	(123–125)
WT1	Wilms tumor 1	AD	(126)
XPO5	Exportin 5	AR	(118)
**METABOLIC, LYSOSOMAL, ENDOCYTIC, AND CYTOSOLIC PROTEINS**			
ALG1	Asparagine-linked glycosylation 1	AR	(127)
CFH	Complement factor H	AR	(128)
DGKE	Diacylglycerol kinase, epsilon	AR	(129)
PMM2	Phosphomannomutase 2	AR	(130)
SCARB2	Scavenger receptor class B member 2	AR	(131)
SGPL1	Sphingosine-1-phosphate lyase	AR	(132)
TTC21B	Tetratricopeptide repeat protein 21B	AR	(133)
ZMPSTE24	Zinc metalloproteinase STE24	AR	(134)

### Treatment of Nephrotic Syndrome Based on Genetic Cause

The vast majority of cases of monogenic nephrotic syndrome do not respond to steroids or other immunosuppressive agents, though a growing number of cases of SSNS are now being attributed to a single gene cause [recently reviewed by (135)]. This response may be explained by recent studies which suggest that in addition to their immunosuppressive effects, corticosteroids act directly on the podocyte actin cytoskeleton to regulate expression of slit diaphragm proteins (136, 137). Similarly, several studies have examined the non-immunomodulatory effects of calcineurin inhibitors (CNIs). CNIs may affect the podocyte in a number of ways, including by altering the intra-renal hemodynamics and directly leading to decreased proteinuria, by stabilizing the actin cytoskeleton through degradation of synaptopodin, and through upregulation of cofilin-1 (138, 139). Despite these hypotheses, in clinical practice very few children with monogenic nephrotic syndrome respond to CNIs. As our understanding of the direct effects of various immunosuppressive agents on the glomerular filtration barrier grows, genetic testing will likely play an increasing role in guiding treatment decisions for patients with SRNS and even with SSNS.

### Genetic Testing as a Biomarker for Prognosis

Genetic testing may be helpful in predicting the timing of progression to ESRD. Bierzynska et al. showed that patients with a genetic form of SRNS progressed to stage 5 chronic kidney disease faster than those without a detected gene mutation (4.75 vs. 6.28 years, *p* = 0.0082) (6). Additionally, genetic testing can provide invaluable information about the risk of disease recurrence following kidney transplantation. As many as half of patients with SRNS will have recurrence of disease following transplantation. Identifying which patients are at risk for recurrence has historically been difficult, however this is improving in the genomic era. It is clear that patients with monogenic NS are less likely to have post-transplant recurrence, however the precise risk remains uncertain (140).

Ding et al., reviewed 150 patients with SRNS who were post kidney transplantation and found that initial steroid sensitivity was the strongest predictor of recurrence. 92.9% who were initially steroid sensitive but went on to develop secondary SRNS had recurrent disease following transplant. Of the patients with genetic SRNS in their study, none developed post-transplant recurrence. Half of the remaining patients (those with primary steroid resistance and without genetic or familial disease) had post-transplant recurrence (141). Bierzynska et al. recently studied patients in a national SRNS cohort and found a 27.8% recurrence rate. 51.7% of patients without a genetic diagnosis and 0% of those with a genetic form of SRNS developed post-transplant disease recurrence (140).

## Conclusions and Future Directions

Childhood idiopathic nephrotic syndrome is a heterogeneous disorder, though our current approach to diagnosis and treatment is a one size fits all method. In this era of individualized medicine, we are in desperate need of clinical and/or laboratory markers that can help better predict a patient's response to therapy, disease course, and post-transplant recurrence risk in children with idiopathic nephrotic syndrome.

At this time, no validated urinary or serum biomarkers exist for the diagnosis, differentiation, or prognostication of steroid sensitive or SRNS, however several candidate biomarkers have shown promising results. A few of these markers have shown exciting results in the discovery phase but have yet to be validated in large patient cohorts. With the advent of large nephrotic syndrome patient registries and biorepositories, it is now possible to test these markers in large cohorts of patients. And while individual biomarkers could be helpful, panels which combine the predictive value of several individual biomarkers may be most likely to achieve clinical significance. Future efforts should focus on the validation of individual and combinations of urinary and serum biomarkers in children and adults with steroid sensitive and SRNS.

While some progress has been made in the identification of urinary and serum biomarkers, the prize for most rapid development certainly goes to genetic biomarkers. In the past few decades, research in this field has exploded, and we have now identified over 50 genes associated with SRNS. Gene panels or whole exome testing can be used to predict whether a patient will respond to steroids or other immunosuppressive medications and whether recurrence of disease following kidney transplantation is likely. With a positive genetic test, a clinician is able to, with a great deal of certainty, counsel a patient regarding potential response to medications and risk for recurrence of disease after transplant. On the contrary, however, a negative genetic test is less useful at this time. The majority of patients with SRNS with negative genetic testing will respond to calcineurin inhibitors or other immunosuppressive medications, however at this time no biomarker exists to determine which patient is likely to respond to which medication. Additionally, around half of patients without a genetic cause will develop recurrent disease following transplant (140). Several studies have proposed clinical criteria which can be used to predict this risk, however no clinical or laboratory biomarker has been able to serve as a clinical tool for this purpose.

It is certainly an exciting time in the field of biomarker development and discovery. Idiopathic nephrotic syndrome was once considered a single entity, but we now understand that this is a heterogeneous group of disorders. We now have genetic tests and hopefully soon will have other biomarkers which can aid in the individualized management of patients with these conditions. Although more work is needed, it is realistic to predict that some of the biomarkers highlighted in this review will be implemented as part of standard of care in the very near future.

## Author Contributions

HS, BM, and MB contributed to the writing, editing, and final approval of the manuscript. BM and HS created the tables.

### Conflict of Interest

MB is a co-inventor on a patent for a biomarker panel to distinguish steroid responsiveness in NS. The remaining authors declare that the research was conducted in the absence of any commercial or financial relationships that could be construed as a potential conflict of interest.
